# Microphthalmia, persistent hyperplastic hyaloid vasculature and lens anomalies following overexpression of VEGF-A_188_ from the αA-crystallin promoter

**Published:** 2007-01-19

**Authors:** Catrin S. Rutland, Christopher A. Mitchell, Muneeb Nasir, Moritz A. Konerding, Hannes C.A. Drexler

**Affiliations:** 1 School of Biomedical Sciences, Medical School, University of Nottingham, Derby Road, Nottingham, United Kingdom; 2 Centre for Molecular Biosciences, School of Biomedical Sciences, University of Ulster, Coleraine, United Kingdom; 3 Department of Obstetrics and Gynaecology, University of Nottingham, City Hospital, Nottingham, United Kingdom; 4 Department of Anatomy, Johannes Gutenberg-University Mainz, Mainz, Germany; 5 Max Planck Institute for Heart and Lung Research, W.G. Kerckhoff Institute, Bad Nauheim, Germany

## Abstract

**Purpose:**

During growth of the embryonic eye, dose- and site-specific expression of heparin-binding growth factors is critical for the formation of an appropriate vascular supply. Overexpression of vascular endothelial growth factor-A_188_ (VEGF-A_188_), a strongly heparin-binding, endothelial-specific mitogen, leads to severe disturbance of vascular and overall ocular morphology. This study aimed to evaluate the effects of VEGF-A_188_ overexpression on growth of ocular tissue components.

**Methods:**

Stereological and immunohistochemical methods were employed to identify the vascular profiles, ocular tissue proportions, and cell types in VEGF-A_188_ transgenic mice and compare them with wild-type mice.

**Results:**

In VEGF-A_188_ transgenic mice, both lens tissue and total ocular volume were reduced, whereas cross-sectional areas of hyaloid blood vessels, retina, iris, and optic stalk tissues were significantly increased compared to wild-type mice. Endothelial and pericyte cell numbers in the hyaloid vasculature of transgenic mice were increased three fold, with pericytes assuming their characteristic extraluminal position.

**Conclusions:**

Overexpression of VEGF-A_188_ in the murine lens results in microphthalmia, in addition to hypertrophy and persistence of the hyaloid vasculature. This is similar to the human disorder persistent hyperplastic primary vitreous (PHPV). The murine model is a useful, experimental paradigm for investigation of this condition.

## Introduction

The concentration and distribution of matrix-bound growth factors is critical during cellular differentiation and for appropriate tissue patterning in embryogenesis [1]. For instance, in the developing eye, retinal differentiation and growth is dependent on signals emanating in a temporally restricted pattern from the primitive lens. Ocular morphology is, therefore, intimately associated with the rapid expansion of the lens, which throughout embryogenesis, is supported by a tightly adherent circulation system termed the hyaloid vasculature (HV) [1]. During this rapid phase of growth, the lens produces a variety of peptide growth factors that serve to support localized tissue expansion and the temporally restricted maintenance of the HV [1]. Among these factors are FGF2, PDGF-β, and VEGF-A [1-3]. In the case of the vascular endothelium, VEGF-A levels and the cellular expression pattern is normally tightly regulated, with modest alterations, resulting in embryonic lethal phenotypes [4-7]. These studies reinforce the critical nature of VEGF-A expression in the development and maintenance of the vascular system. However, tissue-restricted expression of VEGF-A and its major isoforms in the eye, due to the nonlethal nature of resultant phenotypes, allows a fuller appreciation of the consequences of misexpression of isoforms of this critical growth factor, particularly in the pathogenesis of ocular diseases.

During normal murine HV development, VEGF-A is principally secreted by lens epithelial cells located at the lens equator, and transcripts of the gene are downregulated perinatally [8]. VEGF-A_188_, one specific isoform of VEGF-A, is transcribed from all eight exons of the gene and strongly binds heparin-associated residues [9-11]. VEGF-A_188_ is immediately matrix-bound following secretion [12] and is most highly expressed in the lung [13]. During embryonic development, the soluble isoforms of VEGF-A_120_ and VEGF-A_164_ are the major isoforms expressed [13] with lens capsule heparin-sulphate proteoglycans (HSPG) potentially acting as a VEGF-A reservoir [14]. A number of ocular pathologies are characterized by deregulated neovascularization, and these conditions correlate with increased levels of total VEGF-A [15-17] - most specifically the VEGF-A_165_ isoform [1,18]. However, the role of VEGF-A_188_, the most tightly bound VEGF-A isoform, during development and in the pathophysiology of ocular disease remains to be determined.

In this study, we analyze and interpret an ocular phenotype in transgenic mice resulting from lens-specific overexpression of VEGF-A_188_. The evidence from this study supports the hypothesis that the microphthalmia and lens anomalies are a direct result of perturbations in the vascular morphology of the HV, while the retinal hypertrophy may be a direct consequence of the retinal ganglion cell responses to this growth factor. These results have particular relevance for human fetal conditions characterized by ocular vascular abnormalities, such as retinopathy of prematurity (ROP) and persistent hyperplastic primary vitreous (PHPV), establishing the experimental paradigm that vascular malformation can result in the gross ocular pathologies characteristic of these conditions.

## Methods

### Animal model

The transgene construction, genotyping, and analysis of VEGF-A_120_, VEGF-A_164_, and VEGF-A_188_ mice is described elsewhere [19]. In brief, the open reading frame cDNA of murine VEGF-A_188_ was cloned in frame into a CPV2 construct [1], and transgenic mice were derived according to standard methodologies. The mice generated by these methods demonstrated lens-specific expression of the VEGF-A_188_ protein from the αA-crystallin promoter. In our study, adult female C57Bl6J mice (8 weeks old) and heterozygous αA-crystallin-VEGF-A_188_ transgenic males were housed in a 12 h:12 h light-dark schedule and were allowed to mate. T females were examined and the presence of a vaginal plug was defined as embryonic day 0.5 (E0.5; n=19 pregnant dams). The females were euthanized and their gravid uteri were carefully dissected free. After amnionectomy, the fetuses were delivered, euthanized, and eyes were enucleated. One eye from each embryo was fixed in 10% buffered formal saline (BFS; pH 7.4) overnight, subsequently embedded in araldite, and sectioned for stereological analysis. The contralateral eye was fixed in 4% paraformaldehyde for 30 min, embedded in paraffin, sectioned (5 μm) and prepared for either immunohistochemical or TUNEL staining or fixed in 2.5% glutaraldehyde in cacodylate buffer for ultrastructural studies. All experiments adhered to Home Office (National) and institutional guidelines (comparable to those published by the Institute for Laboratory Animal Research, *Guide for the Care and Use of Laboratory Animals*).

### Ocular stereology

A total of five wild-type mice and six transgenic littermates were randomly selected from the total population of collected fetuses. Following fixation, eyes were processed, critically orientated in a mold, and embedded in araldite. Serial sections (0.5 μm thick) were cut at 50 μm intervals through the eye, sections placed onto slides and subsequently stained with 2% toluidine blue. A three-stage stereological analysis was performed to determine (1) ocular volume, (2) tissue and vascular fractions, and (3) vascular morphometry using systematic random sampling [20,21]. Light microscopic images were obtained using an Olympus microscope and electronic images were captured with the aid of an Olympus T4040 digital camera. Each section was visualized and stereological analysis performed with the aid of the "QProdit" computer imaging software (Leica Imaging Systems, Cambridge, UK).

### Ocular volume

The perimeter of each eye section was traced and the area calculated. Cross-sectional areas from individual sections were multiplied by 50 (to take into account that sections were cut at 50 μm intervals) and subsequently summed to determine fetal ocular volume.

### Tissue fraction

Two systematically random views [21] of each eye section were collected, stored, and analyzed with the aid of a 96-point grid layout. The ocular tissues were assigned to one of 11 tissue-type groups based on their location and histological phenotype: retina, lens stroma, cornea, vitreous humor, aqueous humor, iris, lens hemorrhage, lenticular hyaloid vasculature, retinal hyaloid vasculature, optic stalk, and nonocular tissue (includes the sclera and eyelid).

### Vascular morphometry

A photomicrograph of each section containing HV was analyzed by tracing around each blood vessel. Vessels within the "broken lines" were included within the count, whereas vessels crossing the solid lines were excluded. Blood vessel lengths, cross-sectional areas, diameters, and volumes were calculated using the tissue fraction and ocular volume data based on the following formulas:

Vol all BV (mm^3^)= BV fraction in eye x Volume of eye (mm^3^)

Vol of BV (mm^3^)= Vol all BV (mm^3^) x Area of BV (μm^2^)               			Area of BV (μm^2^)

Lv=2xQ/A= Vol of BV x Number of BV x 10^6^    	         	Forbidden lines (cm) x Number of sections

Length of BV in eye (metres)= (Vol of eye (mm^3^)/1000) x Lv    					100

BV Cross-sectional area (μm^2^)= Volume of BV (mm^3^)    				    	Length of BV (metres)/1000

Vol=volume, BV=blood vessel, Lv=amount of blood vessels within sample area

### Measurement of corneal thickness in neonatal mice

Heterozygous male transgenic mice were crossed with wild-type female mice, and the eyes from resulting litters (P2) were enucleated, fixed in formalin overnight, and embedded in araldite. Sections were cut (5 μm thick) through the geometric center of the eye (containing the optic nerve; located using light-microscopy), placed onto microscope slides, and stained with hematoxylin and eosin. The phenotype of each pup was identified from its ocular morphology as either transgenic or wild-type. The number of cell layers in the cornea was counted manually under a final magnification of 200X. A nonparametric Friedman test was utilized to ensure that there were no significant differences between wild-type embryos selected from litters containing VEGF-A_188_ mice, and one-way analysis of variance (ANOVA) with post-hoc testing (SPSS v11.0) was used to compare wild-type and transgenic mouse corneas.

### Transmission electron microscopy

Ocular samples were embedded in Epon Araldite (TAAB, Aldermaston, Berks, UK), and semithin sections (about 0.8 μm thickness) were cut from blocks on a Reichert-Jung Ultracut-E microtome (Leica Microsystems, Nussloch, Germany), stained with 2% toluidine blue, and examined prior to further ultrastructural analysis. Ultrathin sections of gold interference color (about 80 nm thickness) were cut, and contrasted with uranyl acetate and lead citrate. Sections were viewed using a JEOL JEM-1010 transmission electron microscope (JEOL, Tokyo, Japan) at an accelerating voltage of 80 kV. Electronic images were captured on a Kodak Megaplus camera model 1.6i (Kodak, San Diego, CA).

### Scanning Electron Microscopy

Specimens were fixed in Karnovsky's fixative solution (2% paraformaldehyde/2% glutaraldehyde in 0.1 M phosphate buffer; pH 7.4) for 1 h, washed several times with PBS for 15 min each, followed by post fixation with 1% osmium tetroxide in 0.1 M phosphate buffer for 1 h. After rinsing with PBS for a minimum of 15 min, the specimens were dehydrated with a series of graded ethyl alcohols (70% for 15 min, 95% for 15 min. and three changes of 100% for 10 min each). The samples were then dried using hexamethyldisilazane (HMDS; Sigma-Aldrich, UK). After drying, the specimens were mounted on aluminium stubs with adhesive tabs and coated with gold for 3 min using a Polaron (Energy Beam Sciences, Agawam, MA) sputter coater. The specimens were examined on an AMRAY 1000A (Bedford, MA) scanning electron microscope.

### Immunohistochemistry

Immunohistochemical detection of VEGF-A (1:100 dilution in PBS; Santa Cruz Biotechnology, Inc, Santa Cruz, CA), heparan sulfate proteoglycan (HSPG, 1:100 dilution in PBS; Upstate Biotechnology, Charlottesville, VA), macrophage cell surface marker F4/80 (1:150 dilution in PBS; Southern Biotechnology, Birmingham, AL) or α-smooth muscle actin antibodies (1:400 dilution in PBS; Sigma) was carried out according to the manufacturer's instructions using the Vectastain (Burlingame, CA), ABC staining method.

### Statistical Analysis

Statistical comparisons between groups were assessed using a one-way ANOVA with post-hoc testing, with a p<0.05 considered as significant.

## Results

**Figure 1:**
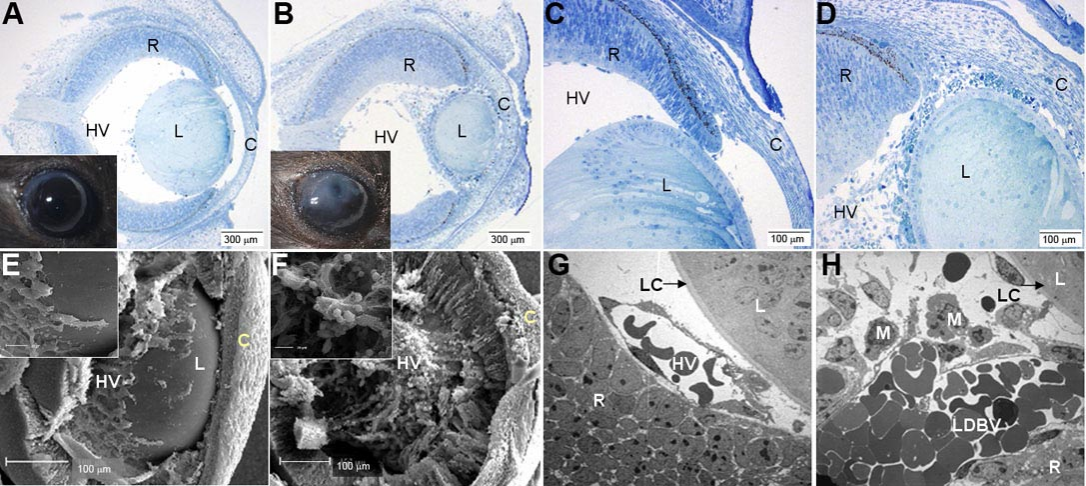
Gross anatomical and microscopic features of VEGF-A_188_ transgenic mice. Representative photomicrographs of toluidine blue-stained sections from E18.5 wild-type (**A**,**C**) and VEGF-A188 transgenic (**B**,**D**) mice. Gross ocular phenotypes of adult wild-type (**A**; inset) and a VEGF-A188 transgenic mouse (**B**; inset), showing conspicuous cataract formation. In VEGF-A188 transgenic mice (B, D), a hypertrophic hyaloid vasculature surrounds a small lens. There is evidence of retinal hypertrophy, particularly in the ganglion cell layer at the top of these micrographs. Scanning electron micrographs of wild-type (**E**) and VEGF-A_188_ transgenic (**F**) mice are shown. The thickened hypertrophic hyaloid vasculature with numerous adherent mononuclear cells in the VEGF-A_188_ transgenic lens (**F**) contrasts with the organized plexus in wild-type mice. Transmission electron micrographs of hyaloid blood vessels in E18.5, wild-type (**G**), and VEGF-A_188_ transgenic mice (**H**). Large diameter vessels(LDBV), with several attendant macrophages (**M**), are conspicuous in VEGF-A_188_ transgenics. In the images, C=cornea, HV=hyaloid vasculature, R=retina, and L=lens.

The gross anatomical features in adult VEGF-A_188_ transgenic mice include microphthalmia and bilateral cataracts (Figure 1A,B; insets). The characteristic feature of sections from eyes of late fetal (E18.5) transgenic mice are fused clusters of vascular channels that are closely apposed to the posterior surface of the lens, retinal dysplasia, and corneal thickening (Figure 1B,D). Lens epithelial cells encircled the lens, the characteristic bowing of lens fiber cells was lost (Figure 1D), and vascular invasion frequently led to intralenticular hemorrhage. Ultrastructural investigations (E15.5) revealed that both endothelial cells and pericytes contributed to a hyperplastic hyaloid vasculature on the posterior pole of the lens, with numerous attendant macrophages (Figure 1E-H).

### Ocular volume and tissue fraction stereology

**Figure 2:**
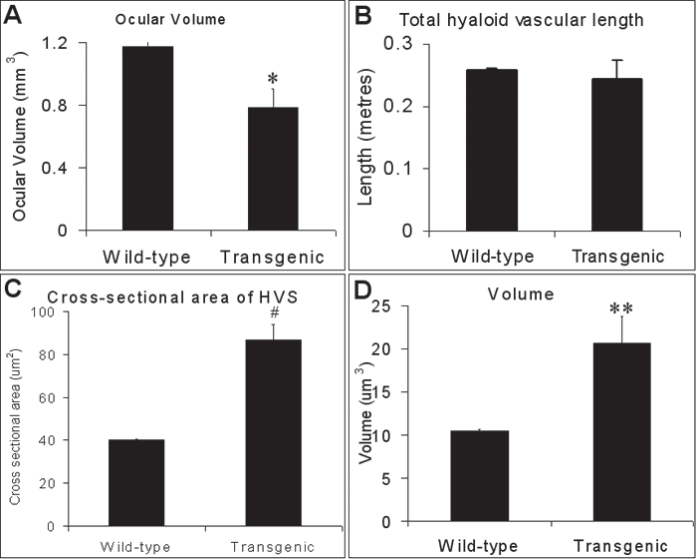
Ocular and hyaloid vascular dimensions in wild-type and VEGF-A_188_ transgenic mice. Total ocular volume (**A**), hyaloid blood vessel length (**B**), cross-sectional area (**C**), and total blood vessel volume (**D**; blood vessel volume=length times cross-sectional area) in E18.5 fetuses from wild-type (n=6) and transgenic (n=5) littermates. Systematic random sampling and stereological methods were used to determine the dimensions of each parameter (see Methods). Statistical analysis was performed using one-way ANOVA with post-hoc testing (SPSS v11.0). The asterisk indicates a p<0.008, the double asterisk indicates a p<0.002, and the sharp (hash mark) indicates a p<0.0001 (wild-type compared to transgenic mice at E18.5). The error bars represent the mean±SEM.

Absolute ocular volumes in VEGF-A_188_ transgenic mice were an average of 33% smaller than their control littermates (mean±SEM=1.1791±0.0287 mm^3^, transgenic=0.7849±0.1180 mm^3^; p<0.008; Figure 2A). In late fetal mice, there are two distinct anatomical regions of the hyaloid vasculature: that surrounding the lens (tunica vascular lentis, TVL, which incorporates the papillary membrane anteriorly), and a retinal component (arteria hyaloidea propria, AHP) on the vitreal surface of the inner limiting membrane [22]. Lenticular vessel proportions (TVL) were increased four fold and retinal vessels (AHP) two fold when compared with wild-type eyes (Table 1), with lens hemorrhage only ever being observed in VEGF-A_188_ transgenic mice (6/6 transgenics; 0/5 controls).

**Table 1:** Proportions and tissue volumes of tissue types in wild-type and VEGF-A_188_ transgenic mice.

**Tissue**		**Proportion of tissue (wild-type; n=5)**	**Proportion of tissue (transgenic; n=6)**	**Increase/ decrease**	**p value**	**Volume of tissue (wild-type; n=5)**	**Volume of tissues (transgenic; n=6)**	**Increase/ decrease**	**p value**
Retina	A	0.3635±0.0185	0.4903±0.0529	1.35 fold increase	0.03*	0.4386±0.0236	0.3572±0.0203	19% decrease	0.04*
Cornea	B	0.0370±0.0064	0.0454±0.0040	-	0.2	0.0430±0.0064	0.0346±0.0047	-	0.3
Optic Stalk	C	0.0066±0.0010	0.0109±0.0013	1.65 fold increase	0.01*	0.0077±0.0012	0.0080±0.0011	-	0.8
Aqueous	D	0.0026±0.0012	0.0085±0.0044	-	0.1	0.4243±0.0292	0.2215±0.0582	48% decrease	0.01*
Vitreous	E	0.3599±0.0240	0.2611±0.0336	-	0.1	0.0031±0.0013	0.0087±0.0046	-	0.3
Hyaloid blood vessels (TVL)	F	0.0037±0.0005	0.0148±0.0019	4 fold increase	0.0001*	0.0043±0.0005	0.0110±0.0013	2.56 fold increase	0.001*
Anterior hyaloidea propria (AHP)	G	0.0052±0.0015	0.0115±0.0020	2.2 fold increase	0.003*	0.0061±0.0016	0.0084±0.0013	-	0.3
Iris	H	0.0193±0.0071	0.0336±0.0152	1.7 fold increase	0.02*	0.0229±0.0047	0.0252±0.0031	-	0.6
Lens stroma	I	0.2023±0.0037	0.0779±0.0041	62% decrease	0.004*	0.2387±0.0113	0.0691±0.0212	71% Decrease	0.001*
Lens hemorrhage	J	0±0	0.0460±0.0085	Not observed in wild-type	0.0001*	0±0	0.0406±0.0126	Not observed in wild-type	0.01*
Total Lens	I+J	0.2023±0.0071	0.1239±0.0029	39% decrease	0.01*	0.2387±0.0113	0.1098±0.0337	54% decrease	0.008*
Total Blood Vessels	F+G	0.0089±0.0017	0.0263±0.0027	2.96 fold increase	0.0001*	0.0104±0.0019	0.0194±0.0019	1.87 fold increase	0.009*

Proportion of total ocular tissue types in wild-type (n=5) and VEGF-A_188_ transgenic (n=6) mice. Values represent mean±SEM. Statistical comparisons between groups were performed using one-way ANOVA, with significant differences designated by an asterisk. Significance was accepted as p<0.05.

The total proportion of lens tissue (stroma plus hemorrhagic tissue) decreased by 39%, whereas lens stromal tissue alone was reduced by 62% in VEGF-A_188_ transgenic mice in comparison to littermate controls. In addition to these conspicuous changes in lens morphology, the fraction of retina, iris, and optic stalk tissue were all significantly increased (Table 1). The fractional proportion of vessels in VEGF-A_188_ transgenic eyes increased in comparison to controls in the AHP (p=0.003), iris (p=0.02), and optic stalk (p=0.01); however, the absolute ocular volumes of these tissue types were not significantly different (Table 1). This indicates that these tissue types were relatively unaffected by the overexpression of VEGF-A_188_, but they occupied a greater volume in a smaller eye. Additionally, despite having a smaller retinal volume, the proportion of AHP and TVL vessels increases (Table 1), particularly in regions around the lens equator (Figure 1). Perilenticular retinal hypertrophy was a consistent feature of VEGF-A_188_ transgenic mice, and general retinal thickening was a consistent finding (both features observed in five out of six transgenic eyes at E18.5; Table 1). To determine whether this vascular and retinal hypertrophy is associated with conspicuous HSPG-bound VEGF-A_188_ in the lens capsule and adjacent retinal surface, we performed immunohistochemical analysis on critically oriented specimens from E18.5 mice.

### Ocular blood vessel stereology

Although blood vessel length was not significantly altered (p<0.58; Figure 2B), the hyaloid vascular cross-sectional area increased two fold (p<0.0001; Figure 2C) in E18.5 VEGF-A_188_ transgenic mice, resulting in a doubling of total vascular volume (p<0.002; Figure 2D). This increase in cross-sectional area of hyaloid vasculature surrounding the lenses of E18.5 VEGF-A_188_ transgenic mice is particularly conspicuous on the posterior and lateral surfaces of the lens (Figure 1B,D,F,H).

### Measurement of corneal thickness in neonatal mice

The non-parametric Friedman test showed that there were no significant differences between wild-type embryos selected from different litters containing VEGF-A_188_ mice (p<0.704; n=45). The number of layers comprising the cornea was significantly increased in the VEGF-A_188_ transgenic mice (15.7±0.99, mean±SEM endothelial cells thick, n=10) in comparison with wild-type mice (12.93±0.50 layers thick, mean±SEM, p<0.033). Despite an increase in the number of corneal layers, the VEGF-A_188_ transgenic eyes did not have an increased corneal thickness in comparison with wild-type mice (p>0.05).

### VEGF immunohistochemistry

**Figure 3:**
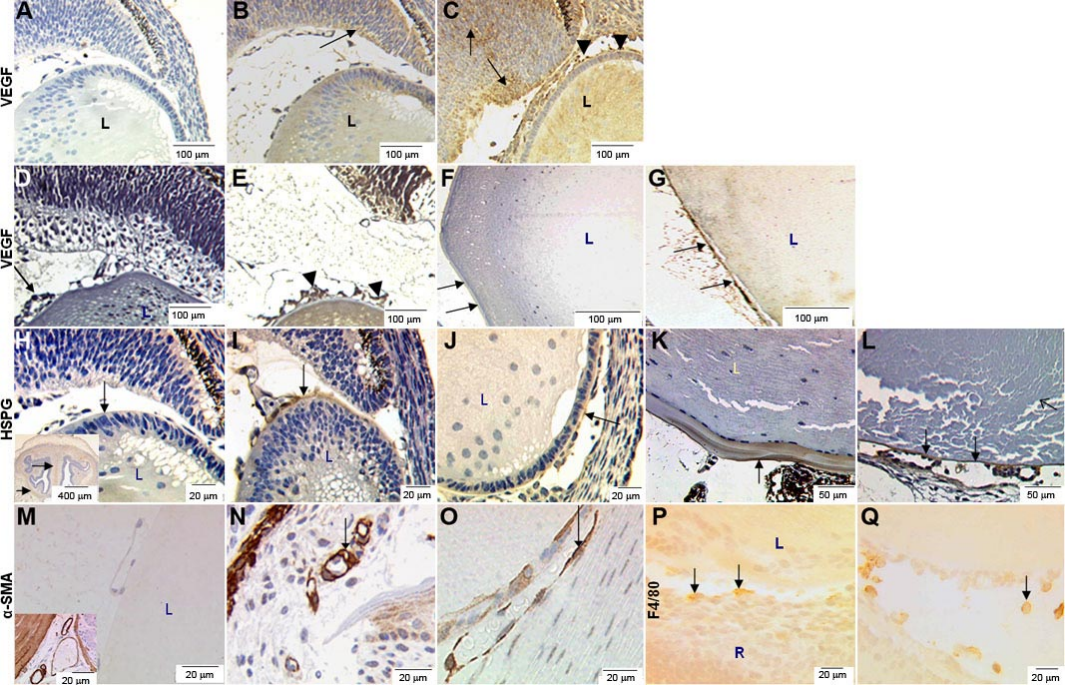
Immunohistochemical detection of VEGF, HSPG, α-SMA, and F4/80 in eyes from wild-type and VEGF-A transgenic mice. Immunohistochemical detection of VEGF-A in wild-type and transgenic mouse eyes at E15.5 (**A**-**C**), P1 (**D**+**E**), and adult (**F**-**G**). **A**; Non-specific IgG serum (control), no discernible background staining is observed. **B**: VEGF-A immunostaining is observed in the wild-type lens (L) and superficial layers of the retina (black arrow) at E15.5. However specific VEGF-A staining was not detected at P1 in the retina, lens, or hyaloid vasculature (**D**). In the adult wild-type eye (**F**), specific staining of the surface of the lens capsule is seen (arrows). Specific staining for VEGF-A is observed in the lens (L), retina (black arrows) and adjacent to the hyaloid vasculature (black arrowheads) at both E15.5 (**C**) and at P1 (**E**) in VEGF-A188 transgenics. In adult VEGF-A188 mice (**G**), prominent staining is observed in the lens capsule (arrows) which appears thinner and (during processing) has separated from the lens structure. Immunohistochemical detection of HSPG (**H**-**L**), α-SMA (**M**-**O**), and F4/80 (**P**,**Q**) in sections from E15.5 (**H**-**J**, **M**-**Q**) and adult (**K**,**L**) mice are shown. **H**: No discernible background staining is observed in control sections (IgG-specific serum). Inset shows an E15.5 mouse brain section, which served as a positive control. **I**: In wild-type mice, specific HSPG staining is observed in the lens capsule and hyaloid vasculature (arrow). **J**: In VEGF-A188 transgenic mice, HSPG staining is observed in the lens capsule (arrow). **K**: HSPG staining is also observed in lens capsule of adult wild-type mice (arrow), with a characteristic laminar pattern. **L**: In adult VEGF-A188 transgenic mice, HSPG staining is seen in the thin lens capsule (arrow) and around persistent hyaloid vasculature (open arrows). Conspicuous staining of peri-vascular smooth muscle cells surrounding blood vessels (arrow) in both E15.5 wild-type (**N**) and transgenic eyes (**O**) was noted. F4/80 immunoreactivity in sections from E15.5 wild- type (**P**) and transgenic mouse eyes (**Q**) revealed cells adjacent to the hyaloid vasculature. In the images, L=lens, R=retina, HV=hyaloid vasculature.

Specific immunohistochemical staining for VEGF-A was observed in sections from the eyes of E15.5, P1 and adult mice (Figure 3). Weak cytoplasmic staining of the retina, lens stroma, and outer lens capsule was observed in wild-type eyes (Figure 3B,D,F). Strong VEGF-A specific immunostaining was observed in the lens, retina, lens capsule, and surrounding the aberrant hyaloid vasculature of E15.5, P1 and adult VEGF-A_188_ transgenic eyes (Figure 3C,E,G, respectively).

### HSPG imunohistochemistry

Immunohistochemical staining for HSPG revealed antibody-specific staining in the lens capsules from the eyes of E15.5 wild-type (Figure 3H,I) and VEGF-A_188_ (Figure 3J) transgenic mice. Lens capsules from E15.5 and adult wild-type mice (Figure 3I,K) were considerably thicker and showed a laminar pattern of HSPG immunostaining in comparison to VEGF-A_188_ mice (Figure 3J,L), where lens capsules were attenuated and intensely HSPG immunoreactive. Staining was also conspicuous around the hyaloid vasculature of transgenic mice (Figure 3J,L).

### α-smooth muscle actin immunohistochemistry

**Figure 4:**
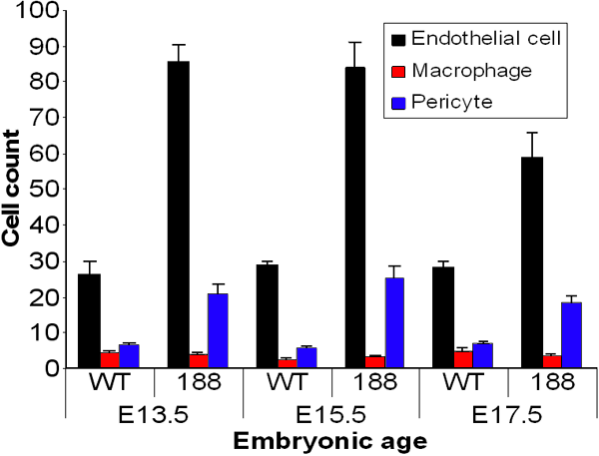
Numbers of ultrastructurally identified cells within the hyaloid vasculature of wild-type and VEGF-A_188_ transgenic mice. Ultrastructural quantitation of cells in the hyaloid vasculature of VEGF-A_188_ transgenics and control littermates. Quantitation of median coronal sections from E13.5, E15.5, and E17.5 VEGF-A_188_ transgenics and control littermates revealed a consistent 3:1 endothelial cell:pericyte ratio. N=4 per group. WT=wild-type. 188=VEGF-A_188_ transgenic mice.

Specific staining for α-smooth muscle actin (α-SMA) was observed in periendothelial cells located on the ablumenal vascular surfaces of capillaries from both wild-type (Figure 3M,N) and transgenic (Figure 3O) mice. In addition, the numbers of pericytes per vessel, as identified by ultrastructural features, was greater in transgenic than littermate controls (Figure 4).

### F4/80 immunohistochemistry

Cells immunostained with the F4/80 antibody, which detects cells from the monocyte/macrophage lineage [23], were observed adhering to the hyaloid vasculatures in both wild-type and VEGF-A_188_ transgenic mice (Figure 3P,Q, respectively).

### Cell types comprising the hyaloid vasculature

The hyaloid vasculature is comprised of three primary cell types: endothelium, specialized macrophages (hyalocytes), and pericytes [24]. Ultrastructural features were used to identify these cell types in representative median sagittal sections from both wild-type and VEGF-A_188_ transgenic eyes. There is an increase in the number of endothelial cells and pericytes in transgenic mice compared with littermate controls at E13.5, E15.5, and E17.5 (P<0.0001, Figure 4). Although there is a 2/3 fold increase in both ECs and pericytes, the ratios of these cell types remained relatively constant throughout fetal development. At E13.5 the ratio of EC:pericytes was 3.88:1 (wild-type) and 4.02:1 (transgenic), compared to E15.5 at 5.08:1 (wild-type) and 3.33:1 (transgenic) and E17.5, where the ratio was 3.89:1 (wild-type) and 3.19:1 (transgenic). Although the trend was toward greater numbers of F4/80 positive cells in transgenic mice, we observed no statistical difference compared to littermate controls (p=0.051).

## Discussion

Microphthalmia in humans is associated with a range of inherited genetic abnormalities, including microphthalmia with linear skin defects (MLS) syndrome [25], Lenz microphthalmia [26,27], and Norrie's disease [28]. Microphthalmia is frequently observed in infants affected with PHPV [29] and this disorder is also commonly associated with premature birth. In the present study, the characteristic ocular phenotype of VEGF-A_188_ transgenic mice is microphthalmia, cataracts, persistent hyperplastic hyaloid vasculature, lens defects, and retinal hyperplasia. The microphthalmia and cataract formation is also a characteristic specifically observed in VEGF-A_188_ mice. This phenotype was not previously reported in mice overexpressing the more labile VEGF-A_165_ isoform when it was overexpressed from the αA-crystalline promoter [1]. In addition, the HV and lens abnormalities are morphologically distinct when either of these two isoforms (i.e., VEGF-A_165_ [1] or VEGF-A_188_ [this study]) is overexpressed - for example, the reduction in lens size is in contrast to observations made when VEGF-A_165_ was overexpressed from the lens [1], where the lens had expanded to fill the space within the vitreal and aqueous compartments. Ash and Overbeek [1] suggest that the enlarged lens observed in the VEGF-A_165_ mice may be due to fluid accumulation in the center of the lens. It is possible that reduction in lens size in the VEGF-A_188_ mice resulted from a lack of nutrients and oxygen obtained from the abnormal TVL and that the disrupted lens fiber cell formation also added to the delayed development of the lens.

A significant reduction in lens size, coupled with cataract formation in VEGF-A_188_ transgenic mice, is similar to the phenotype described in rats that are administered with monosodium-L-glutamate on the 9th and 10th day after birth [30]. Small, cataractous lenses are also associated with the "small eyes" (Sey) phenotype, a semidominant, homozygous-lethal mutation in the mouse [31]. The lenticular hemorrhaging observed in the present study parallels that observed in a patient with congenital cataracts. In the aforementioned study, the authors concluded that the cause of the blood clot was a rupture of the anterior end of the hyaloid artery [32] (attached to the posterior lens surface) and that leakage from hyaloid vessels invading the lens nucleus may be responsible for intralenticular bleeding in such eyes [33].

It is known that VEGF-A is vital to fetal ocular neovascularization [34], particularly during the formation of the HV and retinal vasculatures [1,8,35]. VEGF levels are also often elevated in ocular disorders, where increased vascularization is observed [15,17]. In addition, overexpression of human VEGF-A_165_ under the control of the rhodopsin promoter results in a degenerative phenotype characterized by increased retinal vascularization [18]. The doubling of the hyaloid vascular volumes and cross-sectional areas in VEGF-A_188_ mice is consistent with studies in both VEGF-A_165_ overexpressing tumors [36], where vessel fusion resulted in abnormally large lumen formation and also in normally avascular areas in Japanese quail embryos following injection of nanomolar quantities of VEGF-A_165_ at the onset of vasculogenesis [37]. In VEGF-A_188_ transgenic mice (this study), EC number was significantly increased over that in controls from E13.5-E18.5, and these results are consistent with those described for VEGF-A_165_ mice, where proliferation of presumed endothelial precursor cells juxtaposed to the lens was high during the late fetal period and declined thereafter [1]. The concomitant two to three fold increase of both EC and pericyte numbers during late fetal ocular vascularization of VEGF-A_188_ transgenic mice (this study) is consistent with the hypothesis that the proliferation and cell localization of these cell types are intimately linked. VEGF-A is a known mitogen for pericytes [38], and as both ECs and pericytes express VEGF-R2 they are able to respond to this growth factor [39,40]. Previous studies have also shown that VEGF-A promotes maturation of pericytes within the developing retina [41], and that melanoma cells transfected with VEGF-A cDNA promote a strong proliferative response in both pericytes and ECs [42]. Poor blood vessel development has been suggested to be the result of an insufficient population of mesenchymal cells and pericytes to interact with the overabundance of endothelial cells [1]. This study provides an alternative hypothesis, namely that the pericyte population observed, in both control and VEGF-A_188_ overexpressing mice, increases in tandem with EC number. Hyalocyte (F4/80) positive cells are observed as early as E13.5 in both control and VEGF-A_188_ transgenic mice, in contrast to the low numbers of these cells described in VEGF-A_165_ mice [1]. Hyalocytes are now well known to play a key role in the regression of the hyaloid vasculature, particularly via the expression of Wnt-7b [43]. Our results suggest that the VEGF-A_188_ isoform promotes an increase in hyalocyte numbers, albeit not a statistically significant difference, both within the hyaloid vessel walls and on the ablumenal surface. The role of hyalocytes in the ocular pathology remains to be determined, but the results suggest that as the HV volume increases in VEGF-A_188_ mice, the numbers of hyalocytes do not proportionally increase.

Stereological analysis of VEGF-A_188_ transgenic mice revealed a significantly increased proportion of retina in comparison to wild-type mice, coupled with conspicuous immunolabeling for VEGF in the hypertrophic retina, particularly adjacent to the lens equator. Reduced VEGF-A production in the eye is a cause of retinal thinning [44] and conversely, retinal thickening has been observed in mice with retinal ischemia (associated with increased VEGF-A levels) caused by a surgically increased intraocular pressure [45]. Our data cannot definitively differentiate between retinal hypertrophy resulting from inappropriate neovascularization or as a direct effect of VEGF-A_188_ overexpression on ganglion cell proliferation and differentiation. Overexpression of VEGF-A_188_ from the developing lens also resulted in an increased number of layers in the postnatal cornea of VEGF transgenic mice. One possible explanation for this increased number of EC layers in VEGF-A transgenic corneas is that VEGF-A_188_ transgenic eyes may be subjected to an increased intraocular pressure in relation to the wild-type eye. Several studies have shown that increased intraocular pressure can result in a thickening of the cornea [46-53], however thickening was not observed in our study despite the increase in corneal ECs. Although corneal thickening had not occurred, the properties of the cornea such as protection against noxious agents, biomechanical stability, and structural resiliency as well as the ability to filter out damaging UV light and to aid light refraction may be altered by an increase in density of the cornea. VEGF was originally discovered and termed as vascular permeability factor [54]; therefore, with an increased number of hyaloid blood vessels which are more permeable (as evidenced from the histological and ultrastructural examinations of VEGF-A_188_ transgenic eyes), leakage of serum proteins is highly likely contribute to the ocular pathology we describe.

Previous studies in humans [55] and in mice [1] have demonstrated that VEGF-A is expressed within the lens and supports early fetal lens growth by stimulating the proliferation and migration of angioblasts. In addition to the developmental significance of VEGF-A, a range of ocular pathologies are characterized by inappropriate neovascularization and are coincident with increased levels of VEGF-A. These include proliferative diabetic retinopathy [15,17], age-related macular degeneration [56], retinal neovascularization [18], and retinopathy of prematurity [16]. The immunohistochemical data from this study confirm that VEGF-A is overexpressed within the lens of the transgenic mice and deposited within the lens capsule, where HSPG co-localizes. VEGF-A_188_ has a strong affinity for heparin [12] and the specific lens capsule accumulation of this protein along with HSPG, is consistent with a role for VEGF-A_188_ (and other VEGF-A heparin binding isoforms) in response to wounding. In adult mice and humans, the lens capsule is normally avascular [57], however, following injury such as lens replacement, intense neovascularization surrounding the lens capsule is a common indication for further surgical intervention [58-60]. The presence of HSPG-bound high molecular weight VEGF-A isoforms in the lens may thus prove to be amenable to therapeutic intervention, reducing the incidence of pathological neovascularization.

During normal development of the human eye, the hyaloid vasculature nurtures the developing lens during intrauterine life; in PHPV however, the hyaloid tissue fails to regress and forms a fibrovascular mass behind the lens, resulting in cataracts [29] and bleeding into the vitreous - phenotypes characteristic of the VEGF-A_188_ model. PHPV is a common congenital developmental anomaly of the eye [61], affecting around one in 15,000 live births [62]. Various mammalian species show morphologic features of PHPV, as the disorder has also been reported in cats [63], dogs (where the condition is commonly bilateral [64]), llamas (also bilateral [65]), and in laboratory rats [63], however the etiology of the disorder has not been established. In addition to revealing a role for VEGF-A_188_ in large vessel formation, microphthalmia, lens anomalies and retinal hypertrophy, the VEGF-A_188_ mice described in this study are phenotypically similar to PHPV and should serve as a useful model for preclinical testing of potential therapeutic treatments of this condition.
